# Supplementary material for: The association between health literacy and quality of life of patients with type 2 diabetes mellitus: A cross-sectional study
Source: PLoS One. 2024 Oct 31;19(10):e0312833. doi: 10.1371/journal.pone.0312833 (PMC11527217; doi:10.1371/journal.pone.0312833)
Supplement: Attachment — Submitted filename: Comments for authors health literacy.docx [file pone.0312833.s002.docx]

**The association between health literacy and quality of life of patients with type 2 diabetes mellitus: a cross-sectional study**

**Comments for the Authors**

The study discussed an important issue regarding health literacy and quality of life. The study is well written and easy to follow. Some questions and points were raised during the review process and need to be addressed.

Abstract

Please state the place where the data collection happened.

Please specify what type of diabetes. Consider this throughout the manuscript

Please elaborate on more details including study site, data collection, instruments and data analysis

In the result section: Reword to become more clear ‘The results indicated that increased age was associated with decreased HRQOL

Introduction

Please provide the most recent statistics concerning prevalence of diabetes worldwide, and is there any statistics concerning Jordan.

Is there any site for health literacy in Jordan, the introduction lacks details describing health literacy and HRQOL in Jordan.

Before The Ref [11] Please move The HRQOL paragraph to become before the health literacy paragraph

Material and Methods

Why only Type 2 diabetes were included. I would think that Type 1 would need more education concerning insulin use, could be added as future work

Please specify who collected the data

Have you evaluated the validity and reliability of the study instruments

Regarding EQ-5D REF before ref. [22] mention that this instrument has been used in several earlier studies that evaluated HRQOL among the Jordanian population

Have you conducted a univariate analysis to determine which variables to be included in the regression analysis

Results

Why did the authors use quantile regression and what is the difference between a normal one

Regarding the JDHLQ score How this could be interpreted as high, moderate, low, need improvement, ….etc

The median EQ5-D index score was 0.66 (0.41-0.78). How this could be interpreted as low, moderate, high, …etc

Regarding EQ5-D index scores (Table 3) paragraph make your focus on the association between health literacy and HRQOL, then describe the other associations

Please mention the statistics describing the fitness if the model

Discussion

Similar studies were conducted from your lab please emphasize the novelty and importance of this study.

It would be good to compare your sample with the general population when possible
