# Supplementary material for: The association between health literacy and quality of life of patients with type 2 diabetes mellitus: A cross-sectional study
Source: PLoS One. 2024 Oct 31;19(10):e0312833. doi: 10.1371/journal.pone.0312833 (PMC11527217; doi:10.1371/journal.pone.0312833)
Supplement: Attachment — Submitted filename: Anan Response to reviewers (002).docx [file pone.0312833.s003.docx]

September 24^st^, 2024

Editor-in-Chief, PLOS ONE

**RE: Manuscript Ref. No.: # PONE-D-24-32138- Revisions**

Dear Editor:

We appreciate the helpful suggestions and constructive comments provided by the reviewers. Furthermore, we are pleased that the reviewers found the article interesting and worthy of publication. Moderate revision was requested. Attached please find a revised version of our manuscript (# **PONE-D-24-32138**) entitled: “The association between health literacy and quality of life of patients with type 2 diabetes mellitus: a cross-sectional study”. We have revised the manuscript in response to the reviewers’ comments and believe that our revisions have significantly improved the quality of this manuscript. Below please find our point-by-point response to the comments from Reviewers. The reviewer’s questions are included in black, and our responses are shown in red.

**Reviewer #1:** The study discussed an important issue regarding health literacy and quality of life. The study is well written and easy to follow. Some questions and points were raised during the review process and need to be addressed.

We thank the reviewer for the comments.

Abstract

Please state the place where the data collection happened.

Thank you for your comment and we added “Al Basheer Hospital in Amman, Jordan ”.

Please specify what type of diabetes. Consider this throughout the manuscript

Thank you specified the type of diabetes (type 2 diabetes) where appropriate throughout the manuscript.

Please elaborate on more details including study site, data collection, instruments and data analysis

Thank you. We have included all the requested information

“This cross-sectional study enrolled 400 Type 2 diabetes patients visiting the endocrinology department at the outpatient clinic at Al Basheer Hospital in Amman, Jordan. The data were collected between 1^st^ of August and 28^th^ of December 2023, using the validated Jordanian Diabetic Health Literacy Questionnaire and the EuroQol-5D tool. A quantile regression analysis was conducted to explore the factors associated with health –related quality of life among the study participants. “

In the result section: Reword to become more clear ‘The results indicated that increased age was associated with decreased HRQOL

Done and reworded. “Findings from regression analysis indicated as patients’ age increased, their quality of life scores significantly decreased”.

Introduction

Please provide the most recent statistics concerning prevalence of diabetes worldwide, and is there any statistics concerning Jordan.

Thank you for your comment and we have updated the global statistics and the statistics concerning Jordan.

Is there any site for health literacy in Jordan, the introduction lacks details describing health literacy and HRQOL in Jordan.

Unfortunately, this information is not available. However, we have included information about health literacy among diabetic patients in the Middle East and linked it to the QOL in this group of population

“Growing demands for individuals to take greater responsibility for their health have underscored the need for adequate health education. This issue is particularly important in the Middle East, where the rising prevalence of diabetes has become a critical concern. In a study of 256 patients with type 2 diabetes in Saudi Arabia, 27.3% exhibited marginal health literacy, while 35.5% had inadequate health literacy. Another stuy revealed that only 11% of individuals with type 2 diabetes attending outpatient clinics across the UAE demonstrated adequate health literacy levels. Thus, it is essential to develop policies and strategies that reflect the values and practices of each society. “

Before The Ref [11] Please move The HRQOL paragraph to become before the health literacy paragraph

We thank the reviewer for the comment and we made the changes as requested.

Material and Methods

Why only Type 2 diabetes were included. I would think that Type 1 would need more education concerning insulin use, could be added as future work

Done as requested. We have added the following in the limitations and future research section

The current study was limited to type 2 diabetes patients and future studies assessing health literacy among patients with type 1 diabetes are deemed necessary”

Please specify who collected the data

Thank you for your comment and this is a self-administered data as we specify it in material and methods and added “The inclusion criteria included patients diagnosed with type 2 Diabetes Mellitus (T2DM) for at least one year, being 18 years or older, literate, with the ability to read and write, since the tools used in this study are self-administered and agreeing to participate in the study by providing written informed consent”.

Have you evaluated the validity and reliability of the study instruments.

Thank you for your comment, the present study used a validated tool to assess patients’ health literacy (JDHLQ), and a well-established tool “the EuroQol-5D” to assess patients’ QoL. The Cronbach’s for the two questionnaires were computed to assess their internal consistency. For the thorough validation process of the JDHLQ please refer to

https://www.mdpi.com/2227-9032/12/7/801.

Regarding EQ-5D REF before ref. [22] mention that this instrument has been used in several earlier studies that evaluated HRQOL among the Jordanian population

Thank you for your comment and we added in Jordan before reference [22].

Have you conducted a univariate analysis to determine which variables to be included in the regression analysis

Thank you for your comment, as the sample size is suitable for number of predictors, all the evaluated predictors were included in the regression model

Results

Why did the authors use quantile regression and what is the difference between a normal one

Thank you. Kindly find the rational for using the quantile regression in the statistical analysis section:

“The normality of EQ5-D index scores was assessed using Q-Q plots. Since the data was not normally distributed, nonparametric tests were conducted, along with a quantile regression analysis to examine the association between EQ5-D index scores and various variables, including gender, age, monthly income, marital status, education levels, insurance status, HbA1c, medications (Insulin, Metformin, and DPP-4 inhibitors), and JDHLQ score. “

Regarding the JDHLQ score How this could be interpreted as high, moderate, low, need improvement, ….etc

-Thank you for the comment, the questionnaire did not establish cutoff points, but higher score indicates higher literacy, and as median score was 22 out of a maximum possible score of 32 (68%) this could be considered as moderate health literacy

The median EQ5-D index score was 0.66 (0.41-0.78). How this could be interpreted as low, moderate, high, …etc

--Thank you for the comment, the questionnaire did not establish cutoff points, but higher score indicates higher Qol of the maximum possible score of 1

Regarding EQ5-D index scores (Table 3) paragraph make your focus on the association between health literacy and HRQOL, then describe the other associations

-The paragraph was rearranged as suggested.

Please mention the statistics describing the fitness if the model

-Thank you for the comment, the following was added:” The R^2^ value was 0.24, indicating that 24% of the variance in EQ-5D scores was explained by the model” Discussion

Similar studies were conducted from your lab please emphasize the novelty and importance of this study.

Thank you. Kindly note that this is the first study in Jordan and the region that evaluated the impact of HL on QoL of diabetic patients. This was clarified in the discussion as follows:” The current study is the first study to assess the role of HL as a predictor of QOL among patients with T2DM in Jordan.”

It would be good to compare your sample with the general population when possible

We thank the reviewer for the comments, Unfortunately, no similar statistics concerning diabetics patients in Jordan could be found.

**Reviewer #2:** 1. The study is interesting. However, the methods adopted is not geared to answer the research question. It is suggested that the authors develop a clear framework for DM related HL and QOL assessment. Without basic understanding of HL levels of the patient population in the region of study, the assessment of HL-QOL will generate structural flaws in HL assessment. The following points need to addressed too.

-Thank you for your comment.

Kindly note that the JDHLQ, employed to assess Health-Related Quality of Life (HRQOL) in this study, has been previously evaluated for face, content, and construct validity in the Jordanian population using factor analysis and Rasch analysis techniques. <https://www.mdpi.com/2227-9032/12/7/801>.

Consequently, this earlier validation study has provided valuable insights into Health Literacy (HL) levels among the same demographic as the current research (same sample).

Additionally, this study utilized the well-established EuroQol-5D tool to assess patients’ quality of life, which has been employed in numerous studies within Jordan. Furthermore, the internal consistency of the EuroQol-5D was assessed by calculating Cronbach’s alpha.

The study employed regression analysis, incorporating potential confounders, to identify the adjusted unique impact of Health Literacy (HL) on Health-Related Quality of Life (HRQOL). The literature is abundant with previous publications that have utilized similar methodologies to explore the relationship between HL and HRQOL. Examples of such studies include the following, among many others:

<https://pubmed.ncbi.nlm.nih.gov/38635779/>.

<https://www.elsevier.es/es-revista-revista-colombiana-psiquiatria-english-edition--479-articulo-health-related-quality-life-health-literacy-S253031202100059X>

<https://www.valueinhealthjournal.com/article/S1098-3015(10)60353-1/fulltext>

2. It is not clear of the questionnaire used to assess HL in DM patients is validated and tested for reliability given the fact that the Arab population is not homogenous, and Jordan DM patients could be patients from Palestine, and other neighboring countries.

-Thank you for your comment. The JDHLQ, used to assess Health-Related Quality of Life (HRQOL) in this study, has previously been examined for face, content, and construct validity within the Jordanian population using factor analysis and Rasch analysis technique on the same sample <https://www.mdpi.com/2227-9032/12/7/801>. This consistency in the target demographic enhances the reliability of the findings in the current research.

Additionally, we have included the Cronbach’s alpha to ensure the reliability of the instrument.

3. How was the questionnaire administered for illiterate patients, how were consents obtained from the same study population group?

-Thank you for your comment. Kindly note that all the participants were literate with minimum elementary level of education as shown in Table 1. To make this clear, we have included the following in the inclusion criteria:

“The inclusion criteria included patients diagnosed with type 2 diabetes for at least one year, being 18 years or older, literate, with the ability to read and write, since the tools used in this study are self-administered and agreeing to participate in the study by providing written informed consent”

4. Was the questionnaire categorized into different domains? And how specific is the questionnaire for DM patients HL assessment? It is advised to provide a breakup of DM direct HL assessment and non-DM related HL assessment items. A reliability and validity score for each of those groups will be beneficial.

-Thank you for your comment.

Please note that the JDHLQ includes two diabetes-related domains focused on information and communication. These domains are interrelated and should not be used separately. All items are specifically designed for diabetic patients. To address this comment and clarify the point, we have included the items of the JDHLQ in Table 2. We also added Cronbach’s alphas to demonstrate the instrument's reliability. For further details on this instrument validity, please refer to <https://www.mdpi.com/2227-9032/12/7/801>.

5. How was the questionnaire administered? Was it by a study coordinator or the treating physician? What were the bias in completing the questionnaire?

-Thank you for the comment. The questionnaires were self-reported and the patients were approached by the study researcher, the following was included :” The researchers (S.A and Z.A) briefly stated the study objectives, confidentiality of the collected information, and the participant’s right to withdraw from the study at any time. Additionally, the patients were informed that self-completing the questionnaire would take 10 minutes approximately”.

Regarding the possible biases the following was included in the limitation section:” The findings of the current study are subject to recall and social desirability biases since part of the study results were derived from self-reported data. Furthermore, it might be that participants who were interested in the study's aims were more encouraged to enrol in the study, which may cause selection bias”

6. This is multicenter-multinational study, was ethics approval obtained from the ethics committees of all the participating institutions/countries?

-Thank you for your comment, kindly note that the present study is a single center study. Please find the following details in the methods section: “This study involved 400 patients with type 2 diabetes attending the endocrinology department at the outpatient clinic of Al Basheer Hospital, one of the largest public hospitals in Jordan, located in East Amman and serving a significant number of patients. ”. Additionally, we have added the following to the limitation section:” Moreover, the results were based solely on one hospital in Jordan. However, Al-Basheer Hospital is one of the largest public hospitals in the country and serves a substantial number of patients.”

7. How was the sample size caculated? How many patients with DM withing the inclusion criteria are seen at the study site annually? There is a high probability that the study is underpowered.

-Thank you for your comment. Unfortunately, the annual number of patients who attend the hospital and meet the inclusion criteria is not available. However please note that we have included the following the sample size calculations:

” In order to calculate the minimum sample size required to produce a regression model with adequate statistical power, the 50 + 8P equation [24] was adopted, where P represents the number of predictors. The study examined the association of 11 variables with patients’ EQ-5D scores. Therefore, the minimum required sample size was 138 patients.” This method was applied by previous publications please see the following examples:

<https://www.ncbi.nlm.nih.gov/pmc/articles/PMC11121691/>

<https://pubmed.ncbi.nlm.nih.gov/37883370/>.

8. The results of the study oscillate between HL and QOL. What was the HL rate among the DM patients? Is there a correlation between sociodemographic and HL, and does that impact QOL in DM patients?

-Thank you for your comment. Kindly note that the evaluation of different sociodemographic variables association with HL was not the aim of the present study, this was discussed in a different study please see

<https://www.mdpi.com/2227-9032/12/7/801>.

To evaluate the correlation between different predictors in the regression model, we have calculated multicollinearity using the variance inflation factor VIF, which revealed that all the values were below 3, as shown in the statistical analysis section of the manuscript.

The median for the JDHLQ score was 22 (18-25) out of a maximum possible score of 32 (69%), indicating moderate literacy. The following was added to the discussion section: “The present study revealed that most participants demonstrated moderate proficiency in understanding and communicating diabetes-related information. Improving HL through targeted educational interventions could improve patients’ QoL”

9. The authors need to explore literature very well, a few landmark studies from the middle east allude to very low levels of HL among DM patients, and the challenges in DM care. Designing a health literacy model for patients with diabetes | BMC Health Services Research | Full Text (biomedcentral.com) and BMJ Open 2023 Feb 6;13(2):e069489.doi: 10.1136/bmjopen-2022-069489.

Thank you. We have added the following to the discussion section to describe the challenges in diabetes care and HL levels among the Middle East population

The International Diabetes Federation (IDF) identifies the Middle East as a key region for diabetes prevalence. While standard treatments exist, preventive measures must be tailored to local cultures. Despite shared language and religion, Middle Eastern countries exhibit significant cultural, economic, and healthcare diversity. Additionally, issues like war, forced migration, climate change, and political instability complicate healthcare delivery [26]. Urbanization, socioeconomic development, sedentary lifestyles, and high consumption of fats and sugary foods have all contributed to increasing obesity and diabetes rates, presenting significant challenges for the region [27]. Growing demands for individuals to take greater responsibility for their health have underscored the need for adequate health education. Low health literacy is seen as a significant obstacle to enhancing health outcomes. Research has consistently shown that individuals with low health literacy tend to have inadequate diabetes knowledge, engage in less effective self-management, experience poor blood glucose control, and incur higher healthcare costs[28,29]. This issue is particularly important in the Middle East, where the rising prevalence of diabetes has become a critical concern. In a study of 256 patients with type 2 diabetes in Saudi Arabia, 27.3% exhibited marginal health literacy, while 35.5% had inadequate health literacy [30]. Another study revealed that only 11% of individuals with type 2 diabetes attending outpatient clinics across the UAE demonstrated adequate health literacy levels [31]. Thus, it is essential to develop policies and strategies that reflect the values and practices of each society. There is increasing evidence that links diabetes to a lower quality of life, with health literacy accounting for 47.5% of the variance in health-related QoL among diabetic patients [32]. However, findings on the relationship between poor health literacy and lower health-related QoL in these patients have been inconsistent [33].
