# Supplementary material for: The association between health literacy and quality of life of patients with type 2 diabetes mellitus: A cross-sectional study
Source: PLoS One. 2024 Oct 31;19(10):e0312833. doi: 10.1371/journal.pone.0312833 (PMC11527217; doi:10.1371/journal.pone.0312833)
Supplement: Attachment — Submitted filename: Evaluation of revised manuscript.docx [file pone.0312833.s004.docx]

**Evaluation of revised manuscript**

**Manuscript Number:** PONE-D-24-32138R1

**Manuscript Title:** The association between health literacy and quality of life of patients with type 2 diabetes mellitus: a cross-sectional study

To be accepted for publication in PLOS ONE, research articles must satisfy the following criteria:
1. The study presents the results of original research.

Yes
2. Results reported have not been published elsewhere.

Yes
3. Experiments, statistics, and other analyses are performed to a high technical standard and are described in sufficient detail.

Yes
4. Conclusions are presented in an appropriate fashion and are supported by the data.

yes
5. The article is presented in an intelligible fashion and is written in standard English.

Yes
6. The research meets all applicable standards for the ethics of experimentation and research integrity.

Yes
7. The article adheres to appropriate reporting guidelines and community standards for data availability.

Ye
